# Introduction of Colonic and Fecal Microbiota From an Adult Pig Differently Affects the Growth, Gut Health, Intestinal Microbiota and Blood Metabolome of Newborn Piglets

**DOI:** 10.3389/fmicb.2021.623673

**Published:** 2021-02-04

**Authors:** Renli Qi, Zhuo Zhang, Jing Wang, Xiaoyu Qiu, Qi Wang, Feiyun Yang, Jinxiu Huang, Zuohua Liu

**Affiliations:** ^1^Chongqing Academy of Animal Science, Chongqing, China; ^2^College of Animal Science and Technology, Southwest University, Chongqing, China; ^3^Key Laboratory of Pig Industry Sciences, Ministry of Agriculture, Chongqing, China

**Keywords:** intestinal microbes, microbiota transplantation, metabolome, gut health, piglets, FMT

## Abstract

Microbiota transplantation is a rapid and effective method for changing and reshaping the intestinal microbiota and metabolic profile in humans and animals. This study compared the different influences of the introduction of fecal microbes and colonic microbes from a fat, adult pig in newborn pigs. Both colonic microbiota transplantation (CMT) and fecal microbiota transplantation (FMT) promoted growth and improved gut functions in suckling pigs up to weaning. FMT was more beneficial for body weight gain and body fat deposition in piglets, while CMT was more beneficial for intestinal health and mucosal immunity. 16S rDNA sequence analysis indicated that both CMT and FMT significantly increased the abundances of beneficial or functional bacteria, such as *Lactobacillus* and *Prevotella_2* genera, in the piglets, and reduced the abundances of harmful bacteria, such as *Escherichia–Shigella*. Blood metabolome analysis showed that transplantation, especially FMT, enhanced lipid metabolism in piglets. In addition, while CMT also changed amino acid metabolism and increased anti-inflammatory metabolites such as 3-indoleacetic acid and 3-indolepropionic acid in piglets, FMT did not. Of note, FMT damaged the intestinal barrier of piglets to a certain extent and increased the levels of inflammatory factors in the blood that are potentially harmful to the health of pigs. Taken together, these results suggested that intestinal and fecal microbiota transplantations elicited similar but different physiological effects on young animals, so the application of microbiota transplantation in animal production requires the careful selection and evaluation of source bacteria.

## Introduction

The digestive tracts of humans and animals harbor a large number of microbes (mainly bacteria) that form a stable symbiotic relationship with their host. Gut bacteria acquire nutrients from food debris in the host gut, and changes in gut microbes are closely related to growth, development, metabolism, and disease development in the host (DeVadder et al., 2016; Cheng et al., 2018; Mulders et al., 2018; Pluske et al., 2018; Depommier et al., 2020; Wang et al., 2020). Touching and colonization by microbes in the intestine in the early stage of growth after birth determine the composition of the intestinal microbiota, which plays an important role in gut development and maturity of immune function in animals (Liu et al., 2019; Wang et al., 2019; Guevarra et al., 2020). However, the invasion of harmful bacteria can lead to an imbalance in the intestinal microbiota or a reduction in bacterial diversity, thus restricting development and causing other diseases (Singh et al., 2018; Lobionda et al., 2019; Sun et al., 2019).

Recently, many studies have shown that intestinal microbiota transplantation is a direct, rapid and efficient intervention method for reshaping and reforming the gut microbiota in humans and animals (Cui et al., 2015; McCormack et al., 2018; Brunse et al., 2019; Canibe et al., 2019). Colonization of newborn piglets with intestinal bacteria from sows by fecal microbiota transplantation (FMT) has benefits, including reducing diarrhea and increasing piglet growth (Cheng et al., 2018; Hu et al., 2018; McCormack et al., 2018; Brunse et al., 2019). In addition, some studies using germ-free (GF) animals have shown that transplantation not only introduces gut microbes but also disseminates the metabolic characteristics of donors to recipient animals. For example, transplanting fecal bacteria from lean and fat pigs into GF mice causes corresponding changes in fat metabolism and adipose deposition in the mice (Yan et al., 2016).

Fecal microbiota transplantation has been widely used in many different microbial colonization studies in humans and animals because it is simple to perform. It is clear that the number, composition and diversity of fecal bacteria are different from those of microorganisms in the digestive tract of animals. However, it is unclear whether the transplantation of fecal bacteria and intestinal bacteria will cause different physiological or metabolic changes in recipient animals. Therefore, the present study tried to analyze and compare the different influences caused by FMT or colonic microbiota transplantation (CMT) on recipient animals. We transplanted microbiota from the colon digesta and fresh feces of a fat, healthy, adult pig into dozens of newborn piglets with similar body conditions and then analyzed and compared the growth, blood biochemical indicators, intestinal morphology, intestinal microbiota, and blood metabolome in the recipient piglets after a period of time.

## Materials and Methods

### Ethics Statement

This animal experiment was conducted according to the Regulations for the Administration of Affairs Concerning Experimental Animals (Ministry of Science and Technology, China). The present study was approved by the Ethics Committee of the Chongqing Academy of Animal Science (No. CAAS-2019013).

### Donor Pig

Five candidate donor fat adult pigs used in the current study consumed a regular diet without antibiotics and probiotics for 6 weeks prior to feces collection. Hog cholera virus, porcine parvovirus, porcine circovirus-2, porcine reproductive, respiratory syndrome virus, pseudorabies virus, foot and mouth disease virus, and mycoplasma hyopneumoniae were detected in four of the pigs. One pig in which no pathogen was detected was used as the trial donor. A total of 100 g of fresh feces was collected from the donor pig after 12 h of fasting, and a fecal bacterial suspension was prepared as previously described (Hu et al., 2018). Subsequently, the donor was sacrificed, and 100 g of digesta in the middle colon section were collected in a sterile sampling bag and then prepared as a colonic bacterial suspension according the methods of Li (Li et al., 2020).

### Piglets and Microbiota Transplantation

**FIGURE 1:**
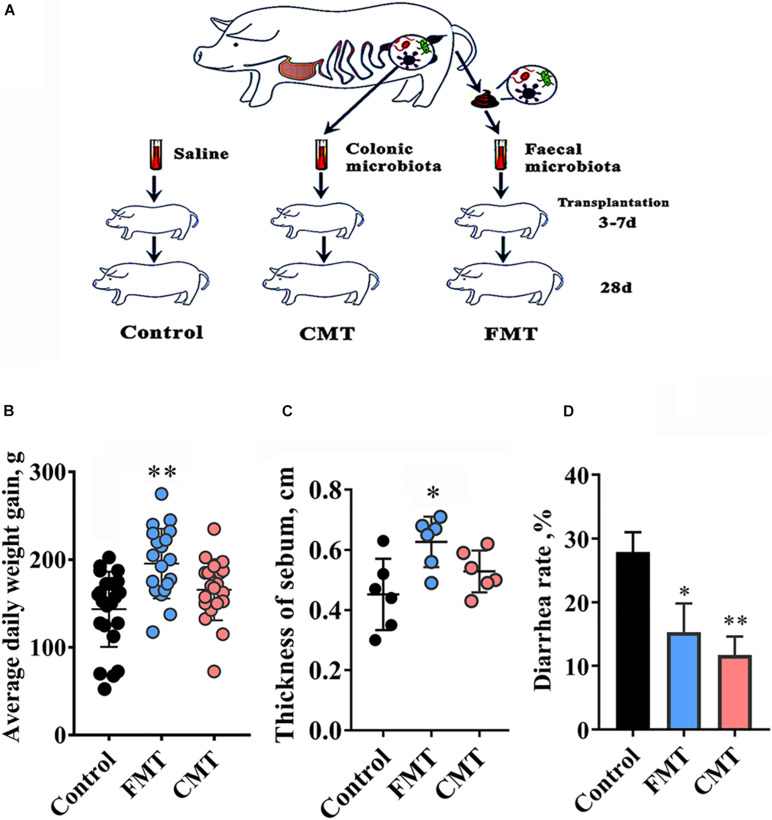
Microbiota transplantation improved growth performance of recipient piglets. **(A)** Schematic of the experimental design. **(B)** The average daily gain of piglets from day 7 to day 28; control group, *n* = 23; FMT group, *n* = 22; CMT group, *n* = 21; **(C)** Neck subcutaneous fat thickness in piglets on day 28 (*n* = 6). **(D)** Diarrhea rate of piglets during the experiment. CMT, colonic microbiota transplantation; FMT, fecal microbiota transplantation. The data are presented as the mean ± S.D. ^∗∗^ Indicates *P* < 0.01, and ^∗^ indicates *P* < 0.05 compared with the control.

A total of nine litters of newborn LR piglets (Landrace × RongChang) with the same birthday and mother parity were used in this animal experiment. Piglets from the nine sows were divided into control, CMT and FMT groups at birth (each group had three pigpens). Six piglets with small birth weights (less than 700 g) were discarded and the control, CMT and FMT groups had 25, 21, and 23 healthy newborn pigs respectively at the beginning of the experiment. In addition, two pigs in the control group, and one pig in the FMT group died during the experimental period. The average birth weight of piglets at the beginning of experiment in control, CMT and FMT groups were 1.11 ± 0.29 kg, 1.11 ± 0.30 kg, 1.12 ± 0.28 kg. The experimental design is illustrated in Figure 1A.

The piglets in the FMT and CMT groups were inoculated orally with 1.0 mL of fecal bacterial suspension (mean count, 5 × 10^8^ CFU/mL) and colonic bacterial suspension (mean count, 8 × 10^8^ CFU/mL), respectively, daily from day 3 to day 7 after born. The piglets in the control group were given the same volume of sterile saline. The three groups of piglets were kept with their mother pigs under the same conditions in three separate houses to avoid the spread and contact of microbes. All piglets were breastfed by sows. In addition, the suckling piglets were offered a common creep feed (Crude protein ≥ 20.0%) ad libitum from day 7 and weaned on day 28. All sows had the same feeding standards.

All piglets in each group were weighed individually at the start (day 3) and end of the experiment (day 28), and average daily gain (ADG) was calculated for all groups. After weighing on day 28, six healthy piglets with approximate average body weight were selected from each group (two pigs per pen) and euthanized by sodium pentobarbital (50 mg/kg body weight). The thickness of subcutaneous fat and the weight of abdominal fat of pigs were measured when the piglets were euthanized.

The number of piglets with diarrhea and the duration of diarrhea were observed and recorded during the experiment. The occurrence of diarrhea was evaluated according to the method of Cheng (Cheng et al., 2019). Diarrhea was defined as stool with a liquid consistency over a minimum of 2 consecutive days. The incidence of diarrhea (%) was calculated as the total number of diarrheal piglets during the period divided by the total number of piglets multiplied by the duration of the trial.

### Sample Collection

A total of 1 g of fresh feces was collected from the piglets after born on days 7, 14, 21, and 28, placed into 2 mL sterile polypropylene centrifuge tubes, frozen in liquid nitrogen and kept frozen at −80°C until DNA extraction. A portion of the feces and colonic digesta from the donor pig were also frozen for DNA extraction. Blood samples, colonic digesta, and intestinal tissue samples were collected when the piglets were euthanized for subsequent biochemical indicator analyses. The midileal and midcolonic segments were sampled and fixed in buffered formalin (10%) at 4°C for morphometric analysis. Ileal samples were also fixed overnight in a 2.5% glutaraldehyde solution at 4°C, and then these samples were treated for observation by electron microscopy. The colonic digesta (from the middle of the colon) were collected for the detection of enzyme activity and short fatty acid content. Feces, blood, digesta, and tissue samples from six pigs for per group (two pigs from each pigpen) were collected for the corresponding detection experiments.

### Examination of Intestinal Morphology

The gut samples were embedded in paraffin wax and sectioned at a thickness of 5 mm on a rotary microtome. Then, the sections were stained with hematoxylin and eosin. Villus height and crypt depth (V/C) were evaluated under a light microscope using a 1/100 ocular scale (Olympus, Japan). The distribution of goblet cells in the intestinal epithelium was observed by light microscopy after the wax was stained with Periodic Acid-Schiff stain.

Clean ileum samples fixed with 2.5% glutaraldehyde were washed with phosphate buffer three times and fixed with 1% OsO4 solution (pH 7.0) for 2 h. After washing with phosphate buffer (0.01 M) three times, the specimens were dehydrated in a graded series of ethanol (30, 50, 70, 80, 90, and 95 %). The segments were transferred to a mixture of alcohol and isoamyl acetate (v: v = 1:1) for 30 min, and then transferred to isoamyl acetate (100%) for 1 h. After being dehydrated with liquid CO_2_ by a critical point dryer (Hitachi Model HCP-2, Japan), the segments were coated with gold palladium and observed under a scanning electron microscope (SEM, Philips Model TM-1000, Japan).

### Biochemical Analysis

Total protein, albumin, triglyceride, total cholesterol, and glucose concentrations in the piglet serum were detected by using a HITACHI 7060 Automatic Analyzer. The growth hormone (#ml002349), insulin (#ml002341), leptin (#ml002355), IgG (ml002328), IgM (#ml002334), TNF-α (#ml002334), IL-1 (#ml002322), IL-6 (#ml002311), and IL-10 (#ml002319) concentrations in the serum samples were evaluated using commercial swine enzyme-linked immune sorbent assay (ELISA) kits (mlbio Co. Ltd., Shanghai, China).

### Western Blotting

Gut tissue samples were lysed in RIPA buffer (Beyotime, Nantong, China), and total protein was extracted. The protein levels of tight junction proteins (Claudin, Occludin, ZO-1/tight junction protein 1) and toll-like receptor (TLR) 2 and 4 were determined by a standard western blotting method with GAPDH as a loading control. Anti-Claudin (#13050-1-AP), anti-Occludin (#13409-1-AP), anti-ZO-1 (#21773-1-AP), anti-TLR4 (#19811-1-AP), and anti-GAPDH (10494-1-AP) primary antibodies were obtained from Proteintech (Wuhan, Hubei, China). An anti-TLR2 (#bs-1919R) primary antibody was obtained from Bioss (Bioss Biotech, Beijing, China).

### Real-Time PCR

Total RNA from the gut mucosa was extracted with an RNAplus kit (TaKaRa, Dalian, China) according to manufacturer’s instructions. cDNA was synthesized via reverse transcription, which was performed with 2 μg of total RNA using a PrimeScript RT reagent kit with gDNA Eraser (TaKaRa, Dalian, China). qPCR was performed using the Q6 qPCR system with SYBR Premix Ex Taq II (TaKaRa). The PCR cycling parameters were as follows: 95°C for 30 s, followed by 40 amplification cycles, each at 95°C for 5 s, then 60°C for 30 s. mRNA levels of Mucin 2 (MUC2) were normalized to the levels of glyceraldehyde-3-phosphate dehydrogenase (GAPDH) as the endogenous control. Primer sequence for *MUC2* gene (Genbank_accession: XM_021082584.1): F-CTGCTCTTGGGCACTATATG; R-CCTG TGACTGCAGAATCAAC and primer sequence for *GAPD*H gene (Genbank_accession: NM_001206359.1): F-CATGGGTAGAATCATACTGGAACA; R-GAAGGTCGGAG TGAACGGAT. Three replicates were performed for all reactions, and the mRNA levels of *MUC2* were expressed as the fold change, which was calculated using the 2^–△△CT^ method.

### Immunohistochemistry

Colon sections were mounted on charged glass slides and processed for immunohistochemical detection of MUC2 using a standard immunoperoxidase technique. A rabbit polyclonal anti-MUC2 antibody was purchased from Proteintech (#27675-1-AP). The antibody was applied at a 1:2000 dilution overnight at 4°C. Diaminobenzidine (Beyotime, Shanghai, China) was used as a chromogen to generate a brown precipitate attributable to its reaction with peroxidase. All slides were counterstained with hematoxylin, rinsed, dehydrated, and mounted with Permount.

### Enzyme Activity Detection

Serum diamine oxidase (DAO) activity (#ml002413) and the activities of digestive enzymes [α amylases(ml076677), β amylases(ml076675), lipase(ml064260), cellulase (ml062909), α glucosinase(ml027530), trypsase(ml076609)] in the colonic digesta were detected using corresponding ELISA analysis kits following the manufacturer’s instructions (mlbio Co. Ltd.).

### 16S rDNA Amplicon Sequencing and Data Analysis

Illumina sequencing of the 16S rDNA gene was performed to characterize the microbial diversity and community composition in fecal samples from the piglets at different times (day 7, day 14, day 21, and day 28). In addition, the colonic contents and feces samples from the donor pig were also assay by the 16s rDNA sequencing. Total genomic DNA from the samples (200 μg) was extracted for amplification using specific primers with the barcode (16S V3 + V4). DNA was amplified by using the 338F/806R primer set (338F: 5′-ACTCCTACGGGAGGCAGCAG-3′, 806R: 5′-GGACTACHVGGGTWTCTAAT-3′). Sequencing was performed by Majorbio Bioinformatics Technology Co., Ltd., Shanghai, China. The sequences were clustered into operational taxonomic units (OTUs) with 97% consistency, and a representative sequence of OTUs was selected. Sequences for each OTU were picked and aligned using Quantitative Insights into Microbial Ecology (QIIME, version 1.9.1). RDP version 11.5 and GreenGenes version 13_8 were used as the reference databases. Alpha diversity analysis (Shannon and Chao1 indexes) was calculated using QIIME. An unweighted unifrac principal coordinate analysis (PCoA) based on OTUs was performed to provide an overview of the microbial diversity and composition in the different pigs. A Co-occurrence network analysis was carried out using Networkx software to reflect the distribution of OTUs in the pigs in different groups. Reconstruction of Unobserved States (PICRUSt) 2.0 was applied to predict the functions of microbiota communities based on the 16S rRNA gene library composition.

### Blood Metabolome and Data Analysis

Blood metabolome analysis was performed using an ultra-performance liquid chromatography (UPLC) system. Chromatographic separation of the metabolites was performed on a ExionLC^TM^ AD system (AB Sciex, United States) equipped with an ACQUITY UPLC BEH C18 column (100 mm × 2.1 mm i.d., 1.7 μm; Waters, Milford, United States). The plasma sample injection volume was 20 μL, and the flow rate was set to 0.4 mL/min. The column temperature was maintained at 40°C. The mobile phases consisted of 0.1% formic acid in water with formic acid (0.1%) (solvent A) and 0.1% formic acid in acetonitrile: isopropanol (1:1, v/v) (solvent B).

After UPLC-TOF/MS analyses, the raw data were imported into Progenesis QI 2.3 software (Nonlinear Dynamics, Waters, United States) for peak detection and alignment. The preprocessing results generated a data matrix that consisted of the retention time (RT), mass-to-charge ratio (m/z) values, and peak intensity. Metabolic features detected at least 50% in any set of samples were retained. The internal standard was used for data QC (reproducibility), and metabolic features in which the relative standard deviation (RSD) of QC > 30% were discarded. Mass spectra of these metabolic features were identified by using the accurate mass. MS/MS fragments spectra and isotope ratio differences were identified by searching the Human metabolome database (HMDB)^1^ and the Metlin database^2^.

Multivariate statistical analysis was performed using the Ropls (Version 1.6.2^3^) R package from Bioconductor on the Majorbio Cloud Platform^4^. Principle component analysis (PCA) using an unsupervised method was applied to obtain an overview of the metabolic data, general clustering, and trends. Orthogonal partial least squares discriminate analysis (OPLS-DA) was used for statistical analysis to determine the global metabolic changes between comparable groups. All of the metabolite variables were scaled to Pareto Scaling prior to conducting OPLS-DA. Variable importance in the projection (VIP) was calculated in the OPLS-DA model. Statistical significance among groups was selected based on VIP values more than 1 and *p*-values less than 0.05. Differential metabolites among the two groups were summarized and mapped into their biochemical pathways through metabolic enrichment and pathway analysis based on a database search (KEGG^5^). The correlation matrix between the lipid metabolites with significant changes and bacterial genera (Top 50) was generated using Spearman’s correlation coefficient by using R (Pheatmap package). Additionally, Procrustes analysis was implemented to reveal the correlation between microbial community and the changed lipid metabolites based on the PCA results.

### Statistical Analysis

All statistical analyses of the data were performed using SPSS 21.0 software (IBM, NY, United States). The data are expressed as the mean ± SD. Differences between groups were analyzed using one-way ANOVA followed by the Tukey–Kramer test. Comparisons of medians between non-normally distributed groups were performed using the Kruskal–Wallis *H* test. *P* < 0.05 was considered statistically significant.

## Results

### Microbiota Transplantation Improved the Growth and Development of Piglets

Figure 1A shows the experimental schematic of this study. Our results showed that FMT and CMT increased average daily gain (ADG, g/d) of piglets to different extents. FMT increased ADG by 36.1% (*P* < 0.01) and CMT increased ADG by 15.5% (*P* = 0.164) in the piglets with transplantation compared to control piglets until weaning (Figure 1B). In addition, the piglets that underwent microbiota transplantation, especially the FMT pigs, which had the most back fat deposition among the three groups of pigs at day 28, were fatter than control pigs (Figure 1C). Transplantation also significantly reduced the rate of diarrhea in piglets during the early stages (Figure 1D).

**FIGURE 2:**
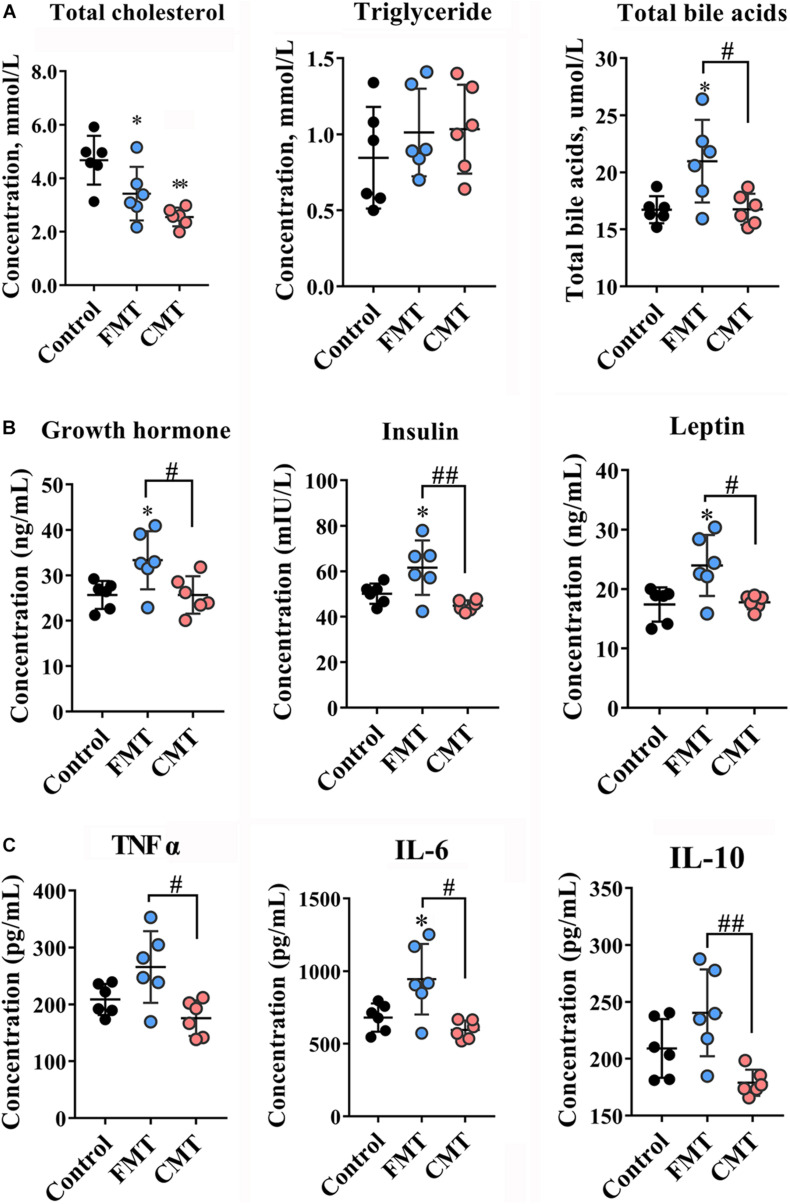
Microbiota transplantation changed blood index of recipient piglets. **(A)** Lipid contents (total cholesterol, triglycerides, total bile acids) in the blood of piglets (*n* = 6). **(B)** Contents of hormones related to growth (growth hormone, insulin, leptin) in the blood of piglets on day 28 (*n* = 6). **(C)** Inflammatory factors (TNF-α, IL-6, IL-10) in the blood of piglets on day 28 (*n* = 6). CMT, colonic microbiota transplantation; FMT, fecal microbiota transplantation. The data are presented as the mean ± SD. ^∗∗^ Indicates *P* < 0.01, and ^∗^ indicates *P* < 0.05 compared with the control; ## indicates *P* < 0.01, and # indicates *P* < 0.05 between the FMT and CMT groups.

We then analyzed the biochemical indicators in the blood of the pigs. The data showed that the total cholesterol (TC) levels dropped significantly after transplantation (*P* < 0.05), while total bile acid (TBA) levels were increased (*P* < 0.05) (Figure 2A). Increasing trends were also observed in triglyceride (TG) and glucose levels in the pigs with transplantations (Figure 2A and Supplementary Figure 1). There were no significant differences in immunoglobulin G (IgG) and IgM, total protein and albumin contents in the different groups of piglets (Supplementary Figure 1). Importantly, FMT, but not CMT, significantly increased the blood levels of growth hormone, insulin, and leptin (Figure 2B, *P* < 0.05) which contributed to the growth of pigs. We also noticed that only FMT caused significant increases in the blood concentrations of inflammatory factors (TNF-α, IL-6, and IL-10) in piglets compared with the other pigs (*P* < 0.05 or *P* < 0.01, Figure 2C).

### Microbiota Transplantation Improved Intestinal Morphology and Functions in Piglets

**FIGURE 3:**
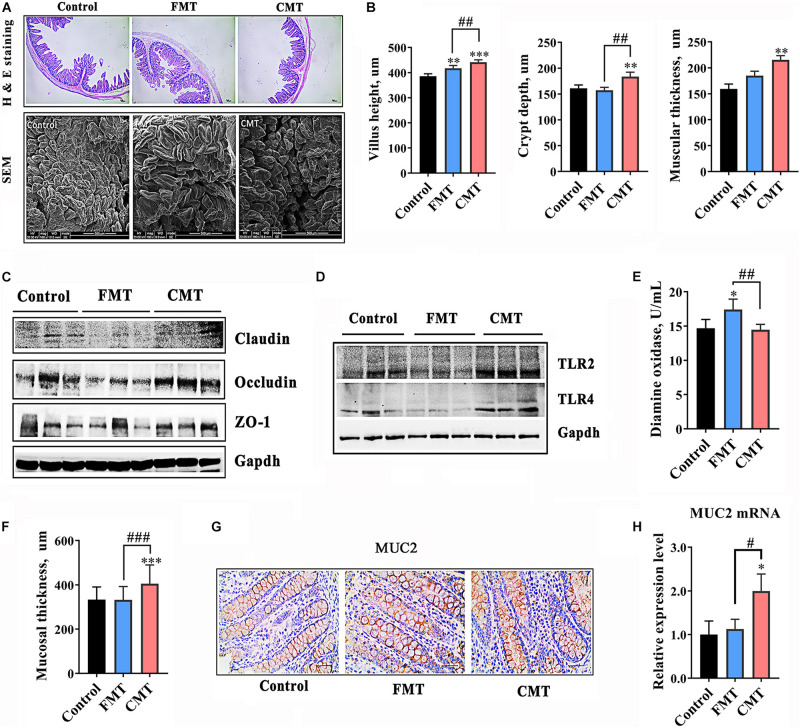
CMT improved development and immune function of the gut in piglets. **(A)** Hematoxylin-eosin staining images (40 × magnification) and scanning electron microscope images (300 × magnification) of ileal sections from piglets on day 28. **(B)** Development index of the ileum (height of ileal villi, depth of ileal crypts, and muscular thickness). **(C)** Protein levels of tight junction proteins in the ilea of piglets. The expression levels of proteins were analyzed by western blotting. **(D)** Protein levels of toll-like receptor 2 and 4 (TLR2 and TLR4) in the ilea of piglets. **(E)** Diamine oxidase content in the blood of piglets. **(F)** Mucosal thickness in the colons of piglets. **(G)** Immunohistochemical staining of the Mucin 2 (MUC2) protein in colonic sections. **(H)** mRNA level of MUC2. The expression level was detected by qRT-PCR (*n* = 6). The data are presented as the mean ± S.D. ^∗∗∗^ Indicates *P* < 0.001; ^∗∗^ indicates *P* < 0.01, and ^∗^ indicates *P* < 0.05 compared with the control; ### indicates *P* < 0.001, ## indicates *P* < 0.01, and # indicates *P* < 0.05 between the FMT and CMT groups.

Next, the effects of FMT and CMT on the development, health and functions of the gut in recipient piglets were evaluated. The piglets treated with CMT exhibited better intestinal morphology (taller villi, *P* < 0.01 and thicker muscularis, *P* < 0.001) and higher protein levels of Occludin (*P* < 0.05) and ZO-1 (*P* < 0.05), two key tight junction proteins (Cheng et al., 2018; Rawat et al., 2020), in the ileum relative to those in the control and FMT pigs (Figures 3A–C and Supplementary Figure 2). CMT also significantly increased the protein levels of Toll-like receptors (TLRs) 2 and 4 in the ileum (*P* < 0.01, Figure 3D and Supplementary Figure 2). However, the tight junction proteins and TLR proteins were decreased in abundance following FMT treatment significantly. To further evaluate the intestinal barrier of pigs, the blood level of diamine oxidase (DAO) (Zhao et al., 2016; Wu et al., 2018) was determined, and the data showed that piglets treated with FMT had a significant increase in the blood level of DAO (*P* < 0.05, Figure 3E).

Compared with the control, CMT significantly increased the thickness of the mucosal layer in the distal intestine of piglets by 23.7% (*P* < 0.001, Figure 3F). Mucin (MUC) 2 exhibited significantly higher protein expression and mRNA expression in the colons of CMT piglets than in the other pigs (*P* < 0.05, Figures 3G,H).

### Microbiota Transplantation Increased Digestive Enzyme Activities in Piglets

**FIGURE 4:**
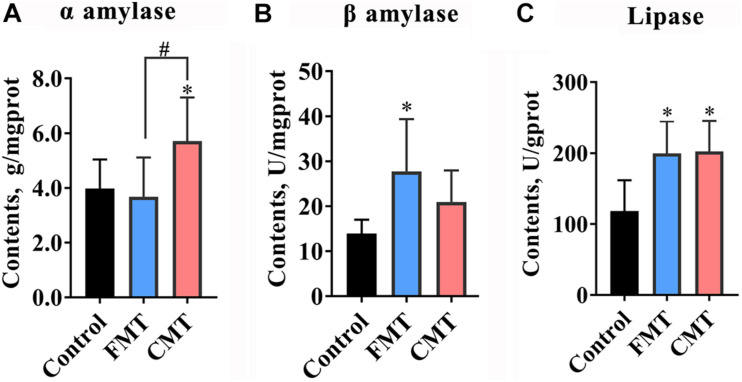
Microbiota transplantation increased the enzyme activities of amylase and lipase in the guts of piglets. **(A)** CMT increase α amylase activity in piglets (*n* = 6). **(B)** FMT increased β amylase in piglets (*n* = 6). **(C)** Both CMT and FMT increase lipase activity (*n* = 6). CMT, colonic microbiota transplantation; FMT, fecal microbiota transplantation. The data are presented as the mean ± SD. ^∗^ Indicates *P* < 0.05 compared with control; # indicates *P* < 0.05 between FMT and CMT.

Figure 4 and Supplementary Figure 2 illustrate the differences in the activities of digestive enzymes in the guts of different groups of piglets. The results showed that the activities of amylases and lipase were significantly changed in the gut of piglets following microbiota transplantation (Figures 4A–C). CMT clearly increased α amylase activity, while FMT significantly increased β amylase activity in the colonic digesta of piglets (*P* < 0.05). Additionally, both FMT and CMT significantly increased the activity of lipase (*P* < 0.05). While a slight increase in the content of cellulase (*P* < 0.1) was observed following the treatments, there were no changes in the activities of α glucosidase and trypsase (*P* > 0.05, Supplementary Figure 3).

### Diversity of Intestinal Microbiota in Different Piglets

**FIGURE 5:**
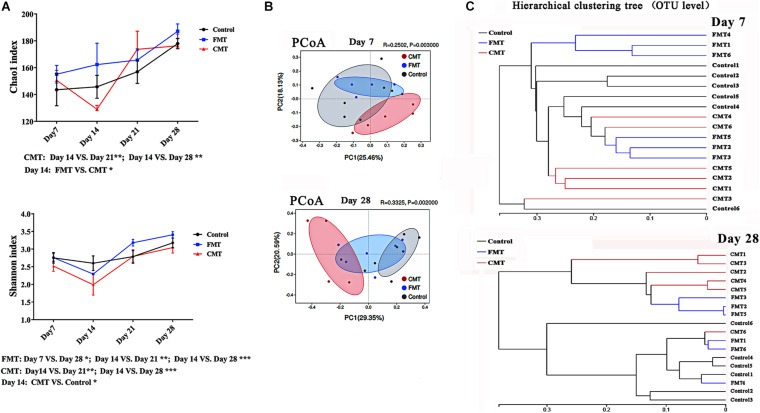
Diversity changes of intestinal microbiota in different piglets. **(A)** Changes in alpha diversity indexes (Chao and Shannon indexes, at the OTU level) of the gut microbiota in piglets after transplantation. * Indicates *P* < 0.05, ** indicates *P* < 0.01, and *** indicates *P* < 0.001; **(B)** Principle coordinate analysis (PCoA) plot based on the OTUs in different piglets on day 7 and day 28. **(C)** Hierarchical clustering analysis of the OTUs in different piglets on day 7 and day 28.

Figure 5A shows the changes in the fecal bacterial alpha diversity (Chao 1 index and Shannon index) in different piglets. Overall, the bacterial diversity increased rapidly in the piglets during early growth. Of note, the pigs in the CMT group had lower Chao and Shannon indexes than control piglets on day 14; however, the diversity indexes were not significantly different among the three groups on day 21 or 28.

Principal coordinate analysis showed that the differences in the microbiota composition became more obvious as the pigs grew after transplantation (Figure 5B). There was also a clear difference between the fecal microorganisms in the pigs from the CMT group and those from the control group until weaning. FMT piglets were more similar to control piglets than to CMT piglets. The results of hierarchical clustering analysis of the microbiota compositions were similar (Figure 5C).

### Microbiota Transplantation Optimized the Structure of the Intestinal Microbiota in Piglets

**FIGURE 6:**
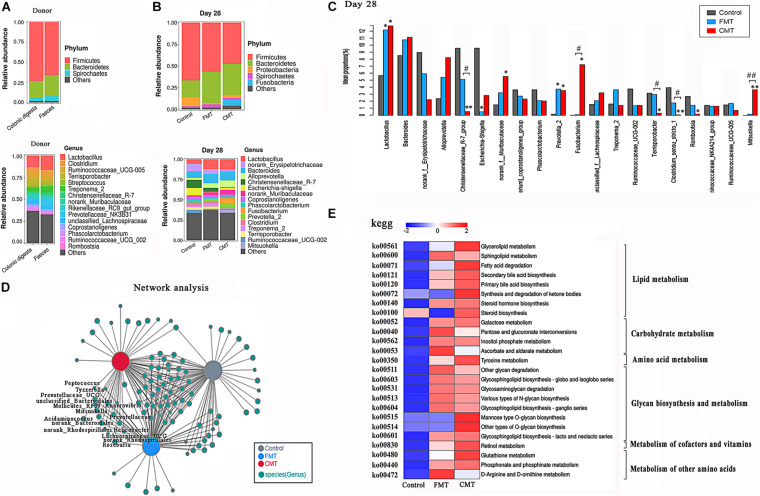
Microbiota transplantation optimized the structure of the intestinal microbiota. **(A)** Bacterial composition profiles of the feces and colonic digesta of the adult donor pig at the phylum and genus levels (*n* = 3). **(B)** Bacterial composition profiles of the feces of the piglets at the phylum and genus levels (*n* = 6). **(C)** The differences in the 20 top bacteria genera with high abundance between different piglets were compared by the Kruskal–Wallis test (*n* = 6); ** indicates *P* < 0.01, and * indicates *P* < 0.05 compared with the control; ## indicates *P* < 0.01, and # indicates *P* < 0.05 between the FMT and CMT groups. **(D)** Co-occurrence network analysis of the gut bacteria (genus level) in different piglets on day 28. **(E)** Comparison of the abundances of KEGG pathways (metabolism, level 3) in different pigs. The functional contributions of the gut microbiota were assessed using the PICRUSt2 tool. Red indicates a high level, and blue indicates a low level.

The overall microbial composition in the three groups of piglets differed at the phylum and genus levels. Figure 6A shows the composition of the bacterial community in the feces and colonic digesta of donor pig. The relative abundance of the *Bacteroides* phylum showed an increasing trend following FMT and CMT treatments (*P* < 0.1), while the relative abundances of the *Firmicutes* (*P* < 0.05) and *Proteobacteria* (*P* < 0.05) phyla were reduced at day 28 after the transplantation (Figure 6B). It seems that the structure of bacterial community of the receptor tended to change to that of the donor pig. Figure 5C shows the differences in the top 20 bacterial genera with high abundances between the different groups of piglets, as analyzed by the Kruskal–Wallis test. Both FMT and CMT increased the relative abundances of common beneficial or functional bacteria such as *Lactobacillus* (Claesson et al., 2007), *Prevotella_2* (Franke and Deppenmeier, 2018), and *Olsenella* (Rosa et al., 2018) while reducing the abundances of pathogenic bacteria such as *Escherichia–Shigella* (Sun et al., 2019) at the genus level (Figures 6B,C). Supplementary Figure 4 illustrates the dynamic changes in the dominant bacteria in the different piglets, as determined by Sankey analysis.

Clearly, FMT and CMT did not have the exact same effects on the bacterial communities in the piglets. CMT alone increased the relative abundances of the *Fusobacterium* and *Mitsuokella* genera and decreased the abundances of the *Christensenellaceae_R-7 group* and *Terrisporobacter* genera. FMT alone increased the relative abundance of the *Treponema_2*, *Marvinbryantia, CAG-873*, and *Blautia.* Linear discriminant analysis Effect Size (LEfSe) was used to identify biomarkers presented as taxons in the three groups (Supplementary Figure 5). CMT piglets were characterized by a higher abundance of *Fusobacterium* and *Mitsuokella* genera while FMT piglets were characterized by a higher abundance of *Prevotella_2* genus. In addition, significant increases in the abundance of many members in the *Prevotellaceae* family, including the *Prevotellaceae* and *Prevotella* genera were observed in the FMT and CMT piglets (Supplementary Figure 6).

A cooccurrence network analysis confirmed that 15 genera, including the *Prevotella_9, Norank-Prevotellaceae, Prevotellaceae_UGC001*, and *Prevotellaceae_UGC003* genera, appeared in the FMT and CMT piglets but were barely detectable in the control piglets (Figure 6D). Furthermore, several other bacteria introduced to piglets by transplantation, such as *Anaerovibrio* (Edwards et al., 2017), *Mitsuolcella* (Zhang et al., 2019), *Oscillospira* (Chen et al., 2020), and *Roseburia* (Kasahara et al., 2018), are known to be related to lipid metabolism, SCFA production, and body fat deposition.

Next, the potential metabolic functions of the microorganisms in the different pigs were predicted by using the PICRUSt2 method. Both CMT and FMT promoted most of the metabolic functions of the gut microbiota in the recipient piglets, especially lipid metabolism, glycan metabolism, and carbohydrate metabolism (Figure 6E).

### Microbiota Transplantation Affected the Blood Metabolome of Piglets

**FIGURE 7:**
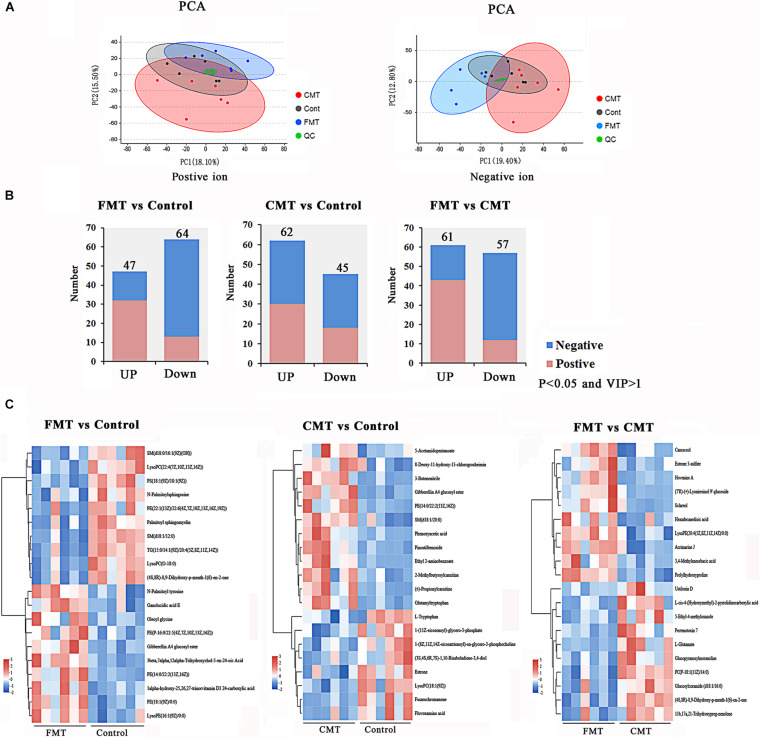
Microbiota transplantation affected the blood metabolome in piglets. **(A)** Principal component analysis (PCA) based on positive and negative blood metabolite profiles. **(B)** The number of differentially expressed metabolites with functional annotations (*P* < 0.05 and VIP > 1) in the three groups of piglets. **(C)** Heatmap demonstrating significantly different metabolites (top 20). Red indicates high abundance, and blue indicates low abundance.

To more deeply investigate the influence of bacterial transplantation on the metabolism of the pigs, the blood metabolites of piglets exposed to different treatments were analyzed by a non-targeted metabolomics approach. PCA was conducted to visualize the differences in the metabolite compositions of the piglets in the three groups (Figure 7A). The blood metabolites were more distinguishable between the three groups of piglets in the negative model than in the positive model.

A total of 299 positive ions and 324 negative ions with functional annotations were identified by metabolome analysis. Of these, 111 (47 up and 64 down), 107 (62 up and 45 down), and 118 (61 up and 57 down) metabolic compounds with functional annotations were significantly different (*P* < 0.05, and VIP > 1) in the three pairwise comparisons (FMT vs. Control, CMT vs. Control, and FMT vs. CMT) (Figure 7B). In Figure 7C, the heatmap shows the top 20 differentially expressed metabolites (*P* < 0.05 and VIP > 1) in the comparisons.

### Microbiota Transplantation Promoted Lipid Metabolism in Piglets

**FIGURE 8:**
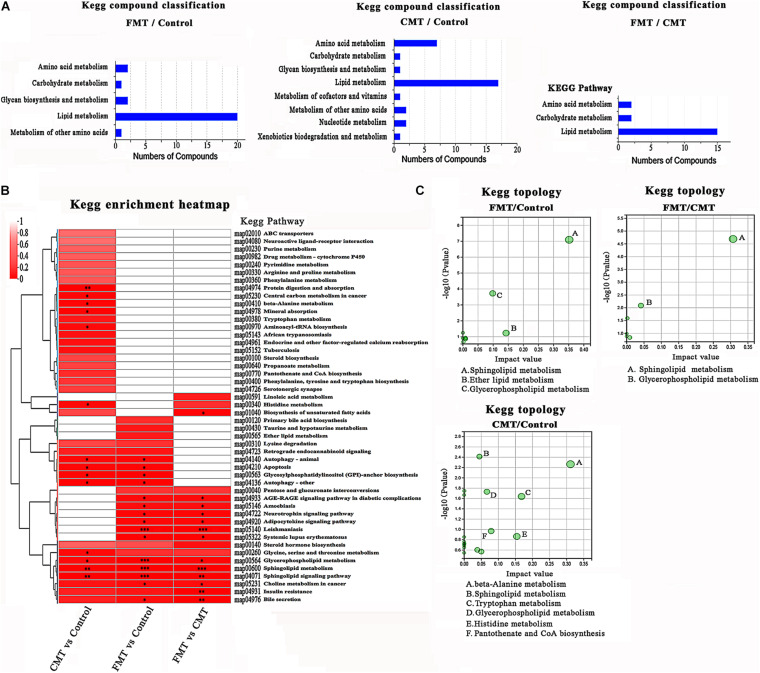
KEGG enrichment analysis of different expressed metabolites. **(A)** KEGG pathway analysis based on the differentially expressed metabolites in the different piglets. **(B)** Clustered heat map of enriched KEGG pathway in the three comparisons. **(C)** KEGG topology analysis of the differentially expressed metabolites in the different piglets.

Similar to the results from the PICRUSt2 functional prediction, the results from the metabolomics analysis revealed that the differentially expressed metabolites in the blood of the pigs were mainly related to lipid metabolism and amino acid metabolism (Figure 8A and Supplementary Figure 7). Up to 88.37, 64.71, and 73.81% of differential metabolites in the three pairwise comparisons were lipids and lipid-like molecules based on Human Metabolome Database (HMDB) compound classification (Supplementary Figure 6). The subclass compound classification results indicated that the dominant differential metabolite compounds were glycerophosphoethanolamines, glycerophosphocholines, phosphosphingolipids, amino acids, and peptides (Supplementary Figure 7B).

The clustered heatmap shows the enriched Kyoto Encyclopedia of Genes and Genomes (KEGG) pathways by differentially expressed metabolite compounds. The altered compounds in FMT piglets and CMT piglets were significantly enriched in lipid metabolism, especially sphingolipid metabolism and glycerophospholipid metabolism. The effects of FMT on the lipid metabolism of piglets were stronger than those of CMT. Notably, changes in amino acid metabolism (especially alanine, histidine, and tryptophan metabolism) were found to be related to CMT treatment by KEGG pathway enrichment and topology analysis, while FMT had a small influence (Figures 8B,C).

In Supplementary Figure 8A, the heatmap shows a total 32 differentially expressed amino acids and its metabolites in the three groups of piglets. Notably, some indole derivatives produced in tryptophan metabolism highly expressed in CMT piglets (Supplementary Figure 8B), such as 3-indoleacetic acid (2.14-fold of control and 1.41-fold of FMT, *P* < 0.05), and 3-indolepropionic acid (2.84-fold of control and 2.39-fold of FMT, *P* < 0.05).

All these results demonstrated that FMT exerted a major influence on the lipid metabolism of piglets; however, CMT significantly affected both lipid metabolism and amino acid metabolism in the piglets.

### Correlation Between Microbiota and Lipid Metabolism

**FIGURE 9:**
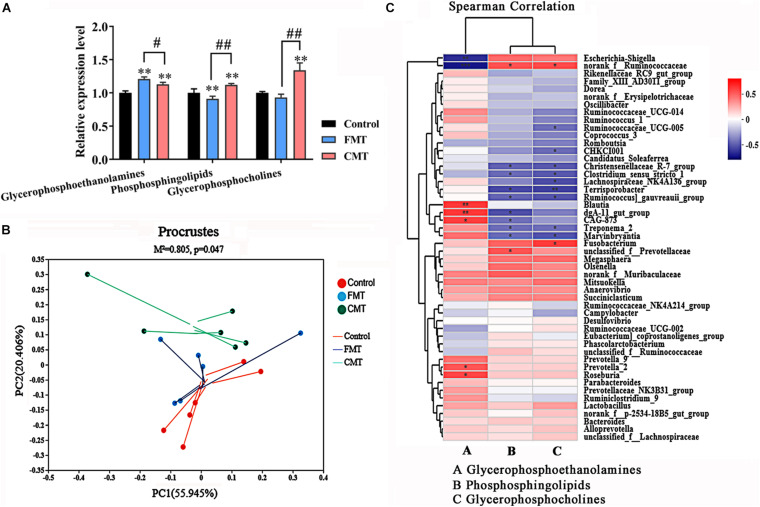
Corrections between intestinal microbiota and lipids metabolism. **(A)** Composition of the three major altered lipid species in the different piglets (*n* = 6); ** indicates *P* < 0.01 with the control, ## indicates *P* < 0.01 and # indicates *P* < 0.05 between the FMT and CMT groups. **(B)** Procrustes analysis shows a significant correlation between lipid profiles and the microbial community. **(C)** Heatmap of Spearman’s correlation coefficients between the intestinal microbiota and the three major altered lipid species. The colors range from blue (negative correlations) to red (positive correlations). Significant correlations are noted by ** *P* < 0.01 and * *P* < 0.05.

Glycerophosphoethanolamines, phosphosphingolipids, and glycerophosphoserines were three dominant lipid species showed the biggest changes in abundance following microbiota transplantation in the pigs. Our data showed that FMT increased the level of glycerophosphoethanolamines and remarkably decreased phosphosphingolipid levels, while CMT increased all three lipid compounds, especially glycerophosphoserines, in piglets (Figure 9A).

Furthermore, Procrustes analysis was carried out to investigate whether the lipid compounds in the piglets were associated with gut microbial composition. As shown in Figure 9B, Procrustes analysis revealed a significant correlation (*M*^2^ = 0.805, *P* = 0.047) between lipid profiles and microbial community profiles in the different piglets. Therefore, we then analyzed the correlations between bacterial abundances (top 50 taxa) and the three changed lipid compounds with Spearman’s correlation coefficients (Figure 9C). The heatmap reflected significant highly positive correlations between the *Blautia*, *CAG-873*, *dgA_11 gut_group, Prevotella-2*, and *Roseburia* genera and the glycerophosphoethanolamines (*P* < 0.05). The *Nonrank_f_Ruminococcaceae* genus was positively correlated with phosphosphingolipids and glycerophosphoserines (*P* < 0.05). In contrast, several genera were highly negatively correlated with phosphosphingolipids and glycerophosphoserines (*P* < 0.05), such as *Treponema_2*, *and Christensenellaceae R-7 group*. These results demonstrate that the transplantation of microorganisms from adult pigs with high fat deposition promotes lipid metabolism in the recipient piglets.

## Discussion

As important livestock, pigs are high-quality meat sources for people in many countries. In addition, pigs are also an important animal model for human medical research since their physiological structure, digestion and metabolic profiles have a high degree of similarity with those of humans (Sangild et al., 2013; Roura et al., 2016). Research on the intestinal microbiota in pigs provides beneficial information for both human health and livestock production.

Recently, the composition and function of the intestinal microbiota of pigs have become more well understood with the widespread application of the high-throughput sequencing, such as metagenomics and metatranscriptomics (Wang et al., 2010; Holman et al., 2017; De Rodas et al., 2018; Gresse et al., 2019). The colonization and succession of intestinal microbes drastically change during the early life of piglets. Diarrhea caused by the invasion of harmful bacteria and resulting intestinal dysplasia is central to early mortality of piglets, which is very detrimental to animal production. After weaning, the intestinal microbiota of pigs gradually stabilizes and adopts more mature functions (De Rodas et al., 2018). Beneficial functional microbes (e.g., *Lactobacillus* and *Prevotella*) have a great potential to contribute to food intake, feeding efficiency, fat accumulation, and muscle growth in pigs. Therefore, improving the intestinal bacterial community and intestinal health in the early growth stages is necessary to promote the growth and health of pigs.

Fecal microbe transplantation is mainly used for the treatment of severe diarrhea and enteritis in the clinic (Cui et al., 2015; Haber et al., 2019). In recent years, some microbial transplantation studies have been carried out in pigs and other domestic animals (Cheng et al., 2018; Geng et al., 2018; McCormack et al., 2018; Brunse et al., 2019; Xiang et al., 2020). The parallel results obtained from these research studies have indicated that early interventions based on microbe transplantation improve the intestinal health of young animals, thereby promoting their growth. The beneficial effects of transplantation are also related to an increase in the relative abundance of beneficial bacteria in the intestine, an increase in the concentration of short-chain fatty acids, the promotion of bile acid metabolism and the reduction of stress in the recipient animal.

Similar to previous works (Cheng et al., 2018; McCormack et al., 2018; Brunse et al., 2019; Xiang et al., 2020), our present study demonstrated that microbiota transplantation following birth significantly promoted the growth of the piglets and improved their intestinal structure, as well as their immune and digestive functions. These beneficial effects in these young pigs are absolutely inseparable from the changes in their gut microorganisms. Both CMT and FMT significantly increased the abundances of beneficial or functional bacteria such as *Lactobacillus*, *Prevotella_2*, and *Roseburia* while also decreasing the abundances of pathogenic bacteria such as *Escherichia–Shigella* and *Erysipelotrichaceae* in the piglets. Among the changed bacteria, the *Prevotellaceae* family (including *Prevotella* and closely related genera) is known to be a dietary fiber fermenter in the gut and a potential biomarker of homeostasis (Kovatcheva-Datchary et al., 2015; Chen et al., 2017; Stanislawski et al., 2019). In addition, several previous studies also reported an association between the abundance of *Prevotella* and the improved ADG in suckling piglets (Kiros et al., 2019; Takahashi and Yamada, 2000). These microbes showed significant increases in the piglets following transplantation that could contribute to the growth, weight gain, and fat deposition of the piglets. In addition, the different transplantations caused changes in a few of unique microbes in the piglets which may reflect a difference in their physiological status.

Importantly, our study emphasized the fact that the physiological responses caused by fecal and colonic microbiota transplantations in animals are similar but also different. Our results indicated that FMT has a more obvious effect on the body weight gain of piglets, while CMT is more conducive to intestinal health and mucosal immunity. We also noticed that FMT causes damage to the intestinal barrier of piglets to a certain extent, including decreased expressions of intestinal tight junction proteins, TLRs (Terán-Ventura et al., 2014) and MUC2 (Cobo et al., 2015). Additionally, FMT also led to an increase in the blood levels of inflammatory factors in piglets, such as TNF-α and IL-6, which may be a potential risk factor for animal production. All of these obvious differences in growth, health, and other physiological responses on the receptors remind us of the need to carefully consider and select the source of the microbiota for transplantation in animal production.

Drastic changes in the intestinal microbiota inevitably result in corresponding changes in the host’s metabolic profiles, which have been well described in previous studies on humans and animals (Yan et al., 2016; Geng et al., 2018). As expected, the metabolic signatures changed after intervention with external bacteria in the early growth stages of pigs in the present study. Both FMT and CMT intensified the lipid metabolism of piglets (especially sphingolipid metabolism), ultimately resulting in increased fat deposition and altered blood lipid contents in the piglets that underwent microbe transplantation relative to the control pigs. The relative abundances of *Blautia*, *CAG-873*, *dgA_11 gut_group, Prevotella-2*, and *Roseburia*, as well as some other gut bacteria, were highly correlated with the changes in lipid compounds. Many of the bacterial genera are well known for their regulatory roles in lipid metabolism and fat deposition in hosts, such as *Blautia* (Ozato et al., 2019) and *Roseburia* (Kasahara et al., 2018).

Therefore, our findings demonstrate that the introduction of the gut microbiota of adult pig facilitates metabolic maturation (especially lipid metabolism) in piglets evidently that providing a potential explanation of how intestinal microorganisms affect the growth and physiological activity of the host. In addition, we observed that only CMT also significantly changed the amino acid metabolism of piglets, especially the metabolism of alanine, histidine, and tryptophan and thus increased the contentions of indole derivatives which primarily come from tryptophan metabolism. These compounds are known to suppress the inflammatory response in humans and animals (Agus et al., 2018; Liang et al., 2018). This also explains how CMT can exert a beneficial effect on gut health in the piglets.

Based on our present study and some previous studies by other groups, we believe that early microbiota transplantation is very beneficial to young animals for accelerating growth and improving intestinal health and functions. Furthermore, we also hope to improve and strengthen the production performance of livestock in the animal husbandry industry in the near future by carrying out better and more efficient microbiota transplantations. Taking into account the different physiological effects caused by the two microbiota transplantations and especially the potential risks brought by FMT in the present study, we must pay more attention to the bacterial source and identify qualified health donors, which will ultimately decide the effectiveness and safety of the transplantation.

Finally, the number of samples used for the analysis of gut microbiota and metabolome may be a limitation of this study. In addition, the separation rearing of the three groups of pigs may affect the gut microbiota, which could interfere with the accuracy of the results to a certain extent.

## Conclusion

To summarize, the results of the present study shows that the early introduction of gut microbes from healthy adult pig to piglets is useful. FMT and CMT exhibited differential positive effects on piglets, including the promotion of growth, reduction of diarrhea, and promotion of digestive function. The difference between the two transplantations was that FMT was more beneficial for body weight gain and body fat deposition, while CMT was more beneficial for intestinal health and mucosal immunity. Both FMT and CMT significantly increased the relative abundance of beneficial bacteria while reducing the relative abundance of harmful bacteria. In addition, transplantation, especially FMT, enhanced lipid metabolism in piglets. Moreover, CMT increased some anti-inflammatory metabolites, such as 3-indoleacetic acid and 3-indolepropionic acid in piglets by promoting amino acid metabolism. Given the differential influences caused by the different transplantations and the potential risk presented by FMT in piglets, such as an increase in inflammatory factors, the application of microbiota transplantation in animal production requires the careful selection and evaluation of source bacteria.

## Data Availability Statement

All the original 16S rRNA sequencing data were submitted to the National Center for Biotechnology Information GenBank Sequence Read Archive database under accession number PRJNA656435.

## Ethics Statement

The animal study was reviewed and approved by the Ethics Committee of the Chongqing Academy of Animal Science.

## Author Contributions

RQ, ZZ, XQ, and JW performed the animal experiments and analyzed the data. QW participated in the data collection. JH assisted with animal experimentation. ZL provided advice in design and performance of experiments. JW wrote the manuscript draft. RQ and ZL critically revised the manuscript. FY supervised the study. All the authors read and approved the final manuscript.

## Conflict of Interest

The authors declare that the research was conducted in the absence of any commercial or financial relationships that could be construed as a potential conflict of interest.

## Footnotes

1 http://www.hmdb.ca/

2 https://metlin.scripps.edu/

3 http://bioconductor.org/packages/release/bioc/html/ropls.html

4 https://cloud.majorbio.com

5 http://www.genome.jp/kegg/

## References

[B1] Agus A. Planchais J. Sokol H. (2018). Gut microbiota regulation of tryptophan metabolism in health and disease. *Cell Host Microbe* 23 716–724. 10.1016/j.chom.2018.05.003 29902437

[B2] Brunse A. Martin L. Rasmussen T. S. Christensen L. Cilieborg M. S. Wiese M. (2019). Effect of fecal microbiota transplantation route of administration on gut colonization and host response in preterm pigs. *ISME J.* 13 720–733. 10.1038/s41396-018-0301-z 30367124 PMC6461782

[B3] Canibe N. O’Dea M. Abraham S. (2019). Potential relevance of pig gut content transplantation for production and research. *J. Anim. Sci. Biotechnol.* 10:55. 10.1186/s40104-019-0363-4 31304012 PMC6604143

[B4] Chen T. Long W. Zhang C. Liu S. Zhao L. Hamaker B. R. (2017). Fiber-utilizing capacity varies in *Prevotella*-versus *Bacteroides*-dominated gut microbiota. *Sci. Rep.* 7:2594. 10.1038/s41598-017-02995-4 28572676 PMC5453967

[B5] Chen Y. R. Zheng H. M. Zhang G. X. Chen F. L. Chen L. D. Yang Z. C. (2020). High Oscillospira abundance indicates constipation and low BMI in the Guangdong Gut Microbiome Project. *Sci. Rep.* 10:9364. 10.1038/s41598-020-66369-z 32518316 PMC7283226

[B6] Cheng C. S. Wei H. K. Wang P. Yu H. C. Zhang X. M. Jiang S. W. (2019). Early intervention with faecal microbiota transplantation: an effective means to improve growth performance and the intestinal development of suckling piglets. *Animal* 13 533–541. 10.1017/S1751731118001611 29983136

[B7] Cheng S. Ma X. Geng S. Jiang X. Li Y. Hu L. (2018). Fecal microbiota transplantation beneficially regulates intestinal mucosal autophagy and alleviates gut barrier injury. *mSystems* 3:e137–18. 10.1128/mSystems.00137-18 30320222 PMC6178585

[B8] Claesson M. J. van Sinderen D. O’Toole P. W. (2007). The genus *Lactobacillus*-a genomic basis for understanding its diversity. *FEMS Microbiol. Lett.* 269 22–28. 10.1111/j.1574-6968.2006.00596.x 17343688

[B9] Cobo E. R. Kissoon-Singh V. Moreau F. Chadee K. (2015). Colonic MUC2 mucin regulates the expression and antimicrobial activity of β-defensin 2. *Mucosal Immunol.* 8 1360–1372. 10.1038/mi.2015.27 25921338 PMC4762903

[B10] Cui B. Feng Q. Wang H. Wang M. Peng Z. Li P. (2015). Fecal microbiota transplantation through mid-gut for refractory crohn’s disease: safety, feasibility, and efficacy trial results. *J. Gastroenterol. Hepatol.* 30 51–58. 10.1111/jgh.12727 25168749

[B11] De Rodas B. Youmans B. P. Danzeisen J. L. Tran H. Johnson T. J. (2018). Microbiome profiling of commercial pigs from farrow to finish. *J. Anim. Sci.* 96 1778–1794. 10.1093/jas/sky109 29635455 PMC6140882

[B12] Depommier C. Hul M. Everard A. Delzenne N. M. De Vos W. M. Cani P. D. (2020). Pasteurized *Akkermansia muciniphila* increases whole-body energy expenditure and fecal energy excretion in diet-induced obese mice. *Gut Microbes* 11 1231–1245. 10.1080/19490976.2020.1737307 32167023 PMC7524283

[B13] DeVadder F. Kovatcheva-Datchary P. Zitoun C. Duchampt A. Bäckhed F. Mithieuxm G. (2016). Microbiota-produced succinate improves glucose homeostasis via intestinal gluconeogenesis. *Cell Metab.* 24 151–157. 10.1016/j.cmet.2016.06.013 27411015

[B14] Edwards H. D. Shelver W. L. Choi S. Nisbet D. J. Krueger N. A. Anderson R. C. (2017). Immunogenic inhibition of prominent ruminal bacteria as a means to reduce lipolysis and biohydrogenation activity *in vitro*. *Food Chem.* 218 372–377. 10.1016/j.foodchem.2016.09.052 27719923

[B15] Franke T. Deppenmeier U. (2018). Physiology and central carbon metabolism of the gut bacterium *Prevotella copri*. *Mol. Microbiol.* 109 528–540. 10.1111/mmi.14058 29995973

[B16] Geng S. Cheng S. Li Y. Wen Z. Ma X. Jiang X. (2018). Faecal microbiota transplantation reduces susceptibility to epithelial injury and modulates tryptophan metabolism of the microbial community in a piglet model. *J. Crohns Colitis* 12 1359–1374. 10.1093/ecco-jcc/jjy103 30010734

[B17] Gresse R. Durand C. F. Dunière L. Blanquet-Diot S. Forano E. (2019). Microbiota composition and functional profiling throughout the gastrointestinal tract of commercial weaning piglets. *Microorganisms* 7:343. 10.3390/microorganisms7090343 31547478 PMC6780805

[B18] Guevarra R. B. Lee J. H. Lee S. H. Seok M. Kim D. W. Kang B. N. (2020). Piglet gut microbial shifts early in life: causes and effects. *J. Anim. Sci. Biotechnol.* 10:1. 10.1186/s40104-018-0308-3 30651985 PMC6330741

[B19] Haber S. L. Raney C. R. K. Larson T. L. Lau J. P. (2019). Fecal microbiota transplantation for recurrent *Clostridioides difficile* infection. *Am. J. Health Syst. Pharm.* 76 935–942. 10.1093/ajhp/zxz078 31361890

[B20] Holman D. B. Brunelle B. W. Trachsel J. Allen H. K. (2017). Meta-analysis to define a core microbiota in the swine gut. *mSystems* 22:e00004–17. 10.1128/mSystems.00004-17 28567446 PMC5443231

[B21] Hu L. Geng S. Li Y. Cheng S. Fu X. Yue X. (2018). Exogenous fecal microbiota transplantation from local adult pigs to crossbred newborn piglets. *Front. Microbiol.* 8:2663. 10.3389/fmicb.2017.02663 29375527 PMC5767267

[B22] Kasahara K. Krautkramer K. A. Org E. Org E. Romano K. A. Kerby R. L. (2018). Interactions between *Roseburia* intestinalis and diet modulate atherogenesis in a murine model. *Nat. Microbiol.* 3 1461–1471. 10.1038/s41564-018-0272-x 30397344 PMC6280189

[B23] Kiros T. G. Luise D. Derakhshani H. Petri R. Trevisi P. D’Inca R. (2019). Effect of live yeast Saccharomyces cerevisiae supplementation on the performance and cecum microbial profile of suckling piglets. *PLoS One* 14:e0219557. 10.1371/journal.pone.0219557 31329605 PMC6645501

[B24] Kovatcheva-Datchary P. Nilsson A. Akrami R. Lee Y. S. Vadder F. D. Arora T. (2015). Dietary fiber-induced improvement in glucose metabolism is associated with increased abundance of *prevotella*. *Cell Metab.* 22 971–982. 10.1016/j.cmet.2015.10.001 26552345

[B25] Li N. Zuo B. Huang S. Zeng B. Han D. Li T. (2020). Spatial heterogeneity of bacterial colonization across different gut segments following inter-species microbiota transplantation. *Microbiome* 8:161. 10.1186/s40168-020-00917-7 33208178 PMC7677849

[B26] Liang H. Dai Z. Liu N. Ji Y. Chen J. Zhang Y. (2018). Dietary L-tryptophan modulates the structural and functional composition of the intestinal microbiome in weaned piglets. *Front. Microbiol.* 9:1736. 10.3389/fmicb.2018.01736 30131777 PMC6090026

[B27] Liu H. Zeng X. Zhang G. Hou C. Li N. Yu H. (2019). Maternal milk and fecal microbes guide the spatiotemporal development of mucosa associated microbiota and barrier function in the porcine neonatal gut. *BMC Biol.* 17:106. 10.1186/s12915-019-0729-2 31852478 PMC6921401

[B28] Lobionda S. Sittipo P. Kwon H. Y. Lee Y. K. (2019). The role of gut microbiota in intestinal inflammation with respect to diet and extrinsic stressors. *Microorganisms* 7:271. 10.3390/microorganisms7080271 31430948 PMC6722800

[B29] McCormack U. M. Curiao T. Wilkinson T. Metzler-Zebeli B. U. Reyer H. Ryan T. (2018). Fecal microbiota transplantation in gestating sows and neonatal offspring alters lifetime intestinal microbiota and growth in offspring. *mSystems* 3:e134–17. 10.1128/mSystems.00134-17 29577087 PMC5864416

[B30] Mulders R. J. de Git K. C. G. Schéle E. Dickson S. L. Sanz Y. Adan R. A. H. (2018). Microbiota in obesity: interactions with enteroendocrine, immune and central nervous systems. *Obes. Rev.* 19 435–451. 10.1111/obr.12661 29363272

[B31] Ozato N. Saito S. Yamaguchi T. Katashima M. Tokuda I. Sawada K. (2019). *Blautia* genus associated with visceral fat accumulation in adults 20-76 years of age. *NPJ Biofilms Microbiomes* 5:28. 10.1038/s41522-019-0101-x 31602309 PMC6778088

[B32] Pluske J. R. Turpin D. L. Kim J. (2018). Gastrointestinal tract (gut) health in the young pig. *Anim. Nutr.* 4 187–196. 10.1016/j.aninu.2017.12.004 30140758 PMC6104527

[B33] Rawat M. Nighot M. Al-Sadi R. Gupta Y. Viszwapriya D. Yochum G. (2020). IL1b increases intestinal tight junction permeability by upregulation of miR200c-3p, which degrades occludin mRNA. *Gastroenterology* 159 1375–1389. 10.1053/j.gastro.2020.06.038 32569770 PMC11752806

[B34] Rosa B. A. Supali T. Gankpala L. Djuardi Y. Sartono E. Zhou Y. (2018). Differential human gut microbiome assemblages during soil-transmitted helminth infections in Indonesia and Liberia. *Microbiome* 6:33. 10.1186/s40168-018-0416-5 29486796 PMC6389212

[B35] Roura E. Koopmans S. J. Lallès J. P. Huerou-Luron I. L. Jager N. Schuurman T. (2016). Critical review evaluating the pig as a model for human nutritional physiology. *Nutr. Res. Rev.* 29 60–90. 10.1017/S0954422416000020 27176552

[B36] Sangild P. T. Thymann T. Schmidt M. Stoll B. Burrin D. G. Buddington R. K. (2013). The preterm pig as a model in pediatric gastroenterology. *J. Anim. Sci.* 91 4713–4729. 10.2527/jas.2013-6359 23942716 PMC3984402

[B37] Singh V. Yeoh B. S. Chassaing B. Xiao X. Saha P. Olvera R. A. (2018). Dysregulated microbial fermentation of soluble fiber induces cholestatic liver cancer. *Cell* 175 679–694. 10.1016/j.cell.2018.09.004 30340040 PMC6232850

[B38] Stanislawski M. A. Dabelea D. Lange L. A. Wagner B. D. Lozupone C. A. (2019). Gut microbiota phenotypes of obesity. *NPJ Biofilms Microbiomes* 5:18. 10.1038/s41522-019-0091-8 31285833 PMC6603011

[B39] Sun J. Du L. Li X. Zhong H. Ding Y. C. Liu Z. H. (2019). Identification of the core bacteria in rectums of diarrheic and nondiarrheic piglets. *Sci. Rep.* 9:18675. 10.1038/s41598-019-55328-y PMC6904459 31822779

[B40] Takahashi N. Yamada T. (2000). Glucose metabolism by *Prevotella* intermedia and *Prevotella* nigrescens. *Oral Microbiol. Immunol*. 15 188–195. 10.1034/j.1399-302x.2000.150307.x 11154402

[B41] Terán-Ventura E. Aguilera M. Vergara P. Martínez V. (2014). Specific changes of gut commensal microbiota and TLRs during indomethacin-induced acute intestinal inflammation in rats. *J. Crohns Colitis* 8 1043–1054. 10.1016/j.crohns.2014.02.001 24566169

[B42] Wang J. Dai D. Zhang H. Wu S. Han Y. Wu Y. (2019). Organic acids modulate systemic metabolic perturbation caused by *salmonella pullorum* challenge in early-stage broilers. *Front. Physiol.* 10:1418. 10.3389/fphys.2019.01418 31803069 PMC6873883

[B43] Wang P. Gao J. Ke W. Wang J. Li D. Liu R. (2020). Resveratrol reduces obesity in high-fat diet-fed mice via modulating the structure and metabolic function of the gut microbiota. *Free Radic. Biol. Med.* 156 83–98. 10.1016/j.freeradbiomed.2020.04.013 32305646

[B44] Wang X. Tsai T. Deng F. Wei X. Chai J. Knapp J. (2010). Longitudinal investigation of the swine gut microbiome from birth to market reveals stage and growth performance associated bacteria. *Microbiome* 7:109. 10.1186/s40168-019-0721-7 31362781 PMC6664762

[B45] Wu T. Zhang Y. Lv Y. Li P. Yi D. Wang L. (2018). Beneficial impact and molecular mechanism of *Bacillus* coagulans on Piglets’ intestine. *Int. J. Mol. Sci.* 19:2084. 10.3390/ijms19072084 30021943 PMC6073773

[B46] Xiang Q. Wu X. Pan Y. Wang L. Cui C. Guo Y. (2020). Early-life intervention using fecal microbiota combined with probiotics promotes gut microbiota maturation, regulates immune system development, and alleviates weaning stress in piglets. *Int. J. Mol. Sci.* 21:503. 10.3390/ijms21020503 31941102 PMC7014131

[B47] Yan H. Diao H. Xiao Y. Li W. Yu B. He J. (2016). Gut microbiota can transfer fiber characteristics and lipid metabolic profiles of skeletal muscle from pigs to germ-free mice. *Sci. Rep.* 6:31786. 10.1038/srep31786 27545196 PMC4992887

[B48] Zhang D. Liu H. Wang S. Zhang W. Wang J. Tian H. (2019). Fecal microbiota and its correlation with fatty acids and free amino acids metabolism in piglets after a lactobacillus strain oral administration. *Front. Microbiol.* 10:785. 10.3389/fmicb.2019.00785 31040835 PMC6476935

[B49] Zhao Y. Zhang Y. Wang G. Han R. Xie X. (2016). Effects of chlorpyrifos on the gut microbiome and urine metabolome in mouse (*Mus musculus*). *Chemosphere* 153 287–293. 10.1016/j.chemosphere.2016.03.055 27018521

