# Supplementary material for: Introduction of Colonic and Fecal Microbiota From an Adult Pig Differently Affects the Growth, Gut Health, Intestinal Microbiota and Blood Metabolome of Newborn Piglets
Source: Front Microbiol. 2021 Feb 4;12:623673. doi: 10.3389/fmicb.2021.623673 (PMC7889522; doi:10.3389/fmicb.2021.623673)
Supplement: Supplementary file 1 [file Data_Sheet_1.pdf]

FIGURE S1. Blood glucose, total protein, albumin, IgG and IgM levels of piglets. The data are presented as the mean ± S.D. n=6. CMT, colonic microbiota transplantation; FMT, fecal microbiota transplantation.

FIGURE S2. Quantification of the intensity of the protein blots in Figure 2. CMT. colonic microbiota transplantation; FMT, fecal microbiota transplantation. The data are presented as the mean ± S.D. n=6. *** indicates $P<0.001$; ** indicates $P<0.01$, and * indicates $P<0.05$ compared with the control; ### indicates $P<0.001$, ## indicates $P<0.01$, and # indicates $P<0.05$ between the FMT and CMT groups.

FIGURE S3. Enzyme activities of cellulose, α glucosinase, and trypsase in the colon of piglets. The data are presented as the mean ± S.D. n=6. No significant differences on the enzymes between the piglets. CMT, colonic microbiota transplantation; FMT, fecal microbiota transplantation.

FIGURE S4. Dynamic changes in dominant bacteria in the different piglets by the sankey analysis. CMT. colonic microbiota transplantation; FMT, fecal microbiota transplantation.

FIGURE S5. Linear discriminant analysis (LDA) effect size (LEfSe) of microbiota in different pigs. LDA score > 4.0. CMT. colonic microbiota transplantation; FMT, fecal microbiota transplantation.

FIGURE S6. Heatmap indicating the differences in the *Prevotellaceae* family and related genera in different piglets. Red indicates high abundance, and blue indicates low abundance. CMT. colonic microbiota transplantation; FMT, fecal microbiota transplantation.

FIGURE S7. Compound classification of the differentially expressed metabolites by the HDMB database screening. A. Superclass level; B. Subclass level. CMT. colonic microbiota transplantation; FMT, fecal microbiota transplantation.

FIGURE S8. Differentially expressed amino acids and its metabolites. A. Heatmap indicating the differences in amino acids metabolites; B. The differences in indole derivatives in the three groups of pigs. CMT. colonic microbiota transplantation; FMT, fecal microbiota transplantation. The data are presented as the mean ± S.E. n=6.

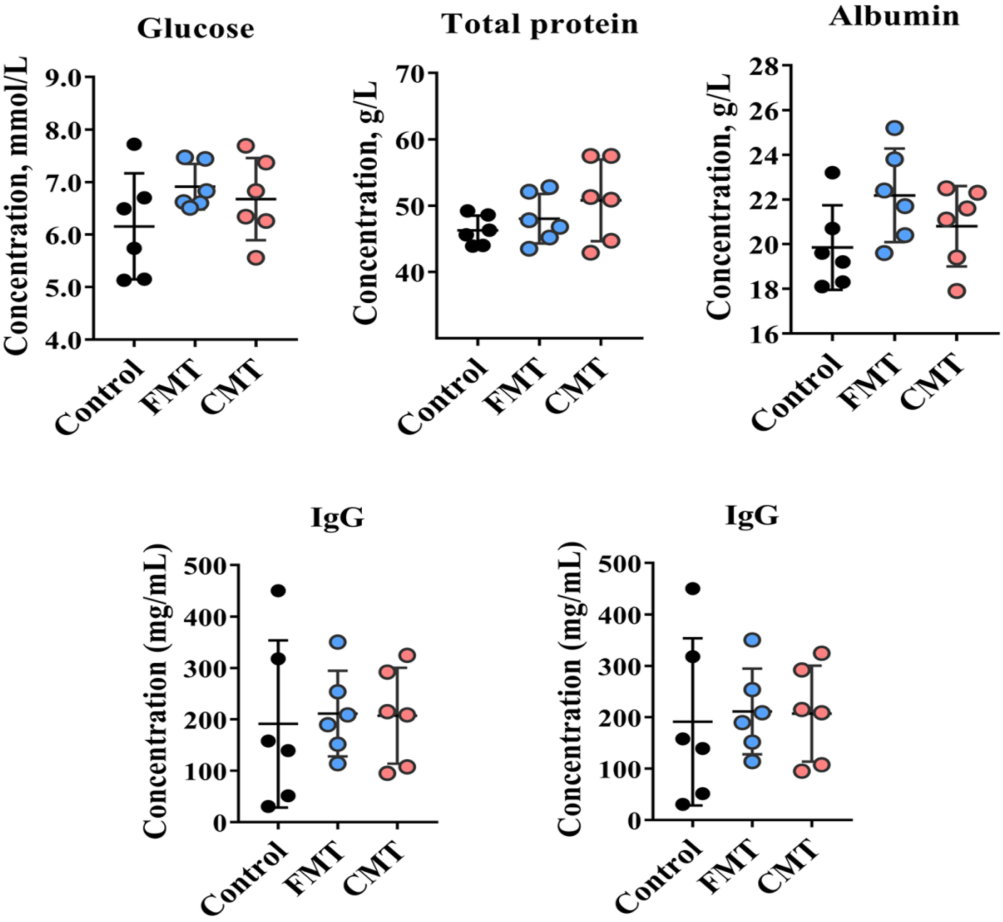
Glucose
Concentration, mmol/L
9.0
8.0
7.0
6.0
5.0
4.0
Control
FMT
CMT
Total protein
Concentration, g/L
70
60
50
40
Control
FMT
CMT
Albumin
Concentration, g/L
28
26
24
22
20
18
16
Control
FMT
CMT
IgG
Concentration (mg/mL)
500
400
300
200
100
0
Control
FMT
CMT
IgG
Concentration (mg/mL)
500
400
300
200
100
0
Control
FMT
CMT

S Fig.1

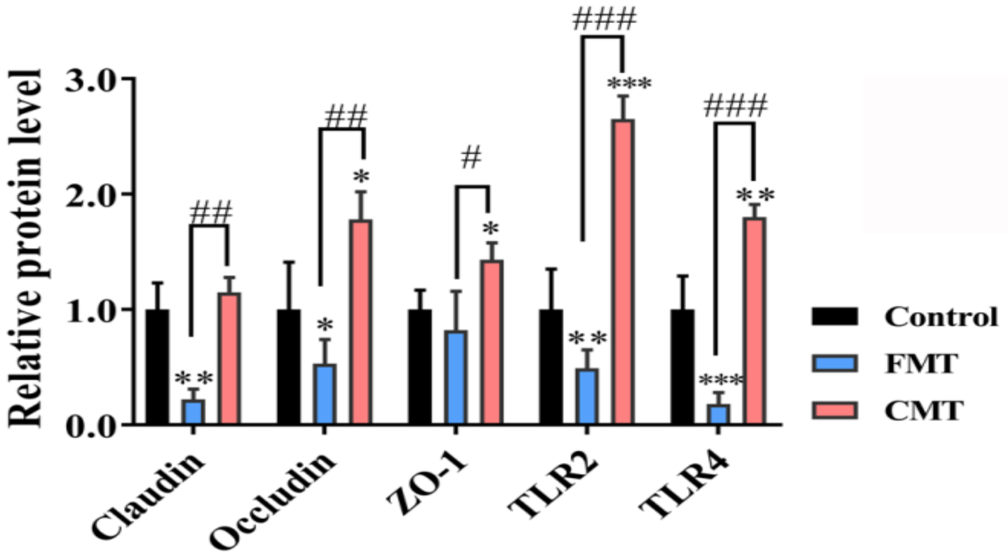

Relative protein level
3.0
2.0
1.0
0.0
Claudin
Occludin
ZO-1
TLR2
TLR4
Control
FMT
CMT
##
##
#
###
###
**
*
*
*
**
***
***
**

S Fig.2

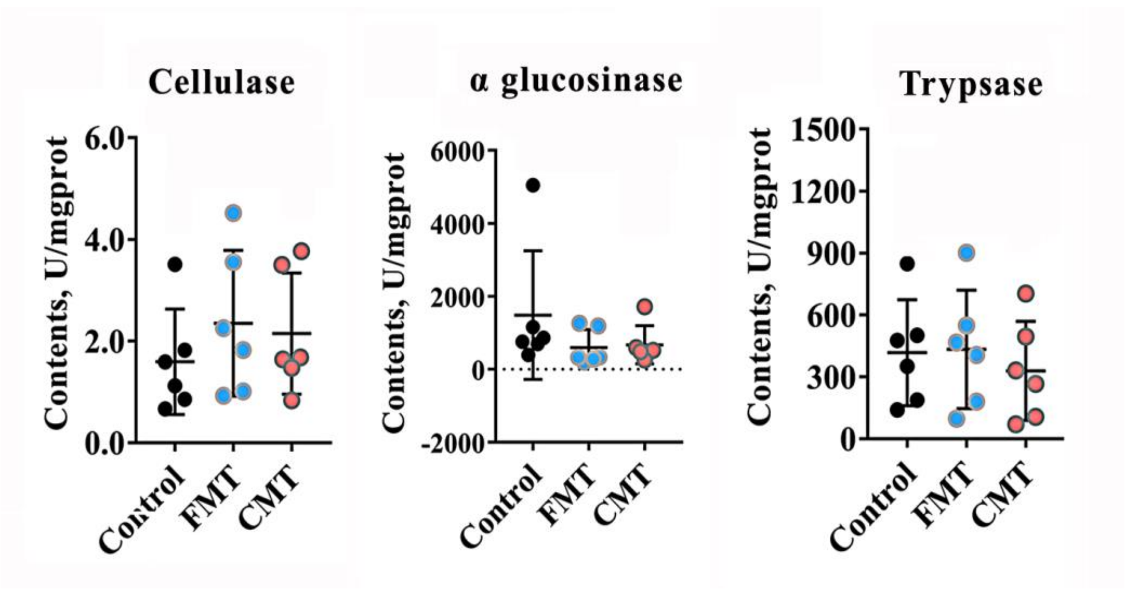
Cellulase
Contents, U/mgprot
6.0
4.0
2.0
0.0
Control
FMT
CMT
α glucosinase
Contents, U/mgprot
6000
4000
2000
0
-2000
Control
FMT
CMT
Trypsase
Contents, U/mgprot
1500
1200
900
600
300
0
Control
FMT
CMT

S Fig.3

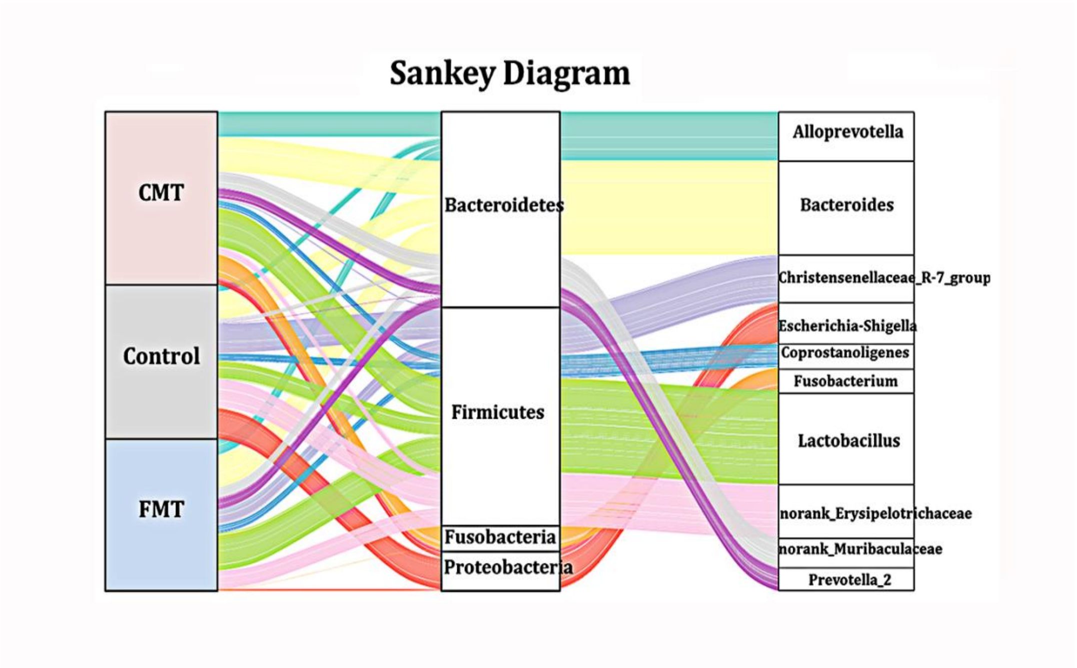
Sankey Diagram
CMT
Control
FMT
Bacteroidetes
Firmicutes
Fusobacteria
Proteobacteria
Alloprevotella
Bacteroides
Christensenellaceae_R-7_group
Escherichia-Shigella
Coprostanoligenes
Fusobacterium
Lactobacillus
norank_Erysipelotrichaceae
norank_Muribaculaceae
Prevotella_2

S Fig.4

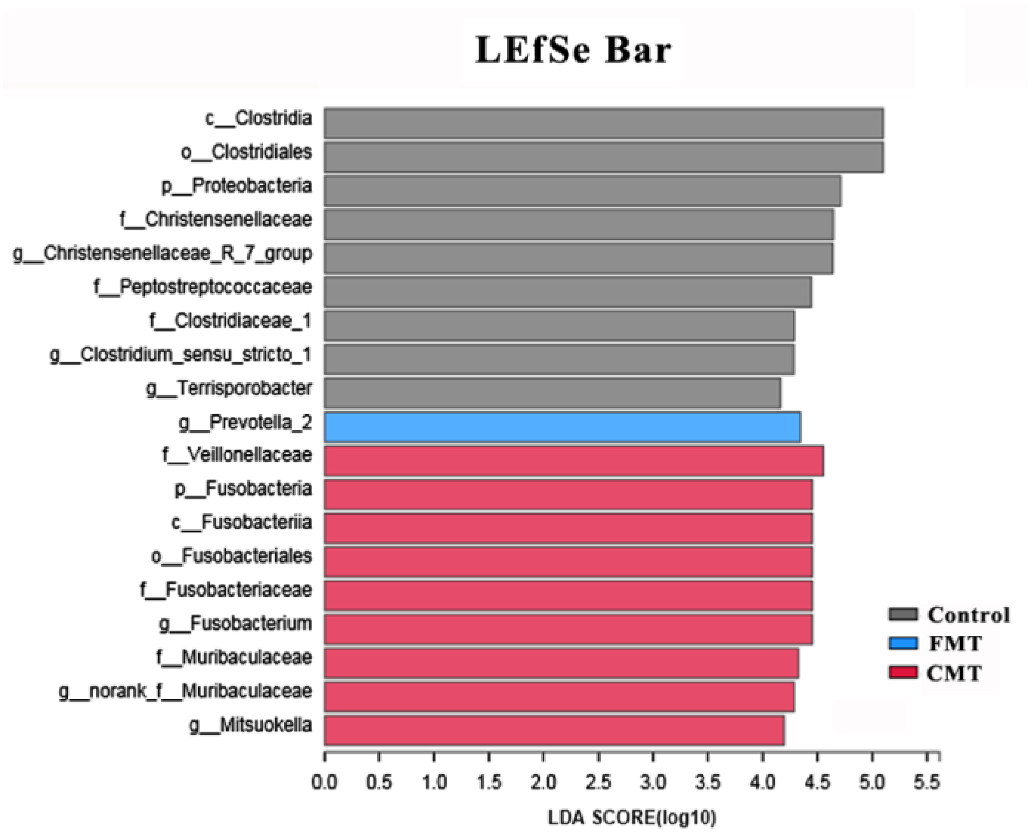
LEfSe Bar
c__Clostridia
o__Clostridiales
p__Proteobacteria
f__Christensenellaceae
g__Christensenellaceae_R_7_group
f__Peptostreptococcaceae
f__Clostridiaceae_1
g__Clostridium_sensu_stricto_1
g__Terrisporobacter
g__Prevotella_2
f__Veillonellaceae
p__Fusobacteria
c__Fusobacteriia
o__Fusobacteriales
f__Fusobacteriaceae
g__Fusobacterium
f__Muribaculaceae
g__norank_f__Muribaculaceae
g__Mitsuokella
0.0 0.5 1.0 1.5 2.0 2.5 3.0 3.5 4.0 4.5 5.0 5.5
LDA SCORE(log10)
Control
FMT
CMT

S Fig.5

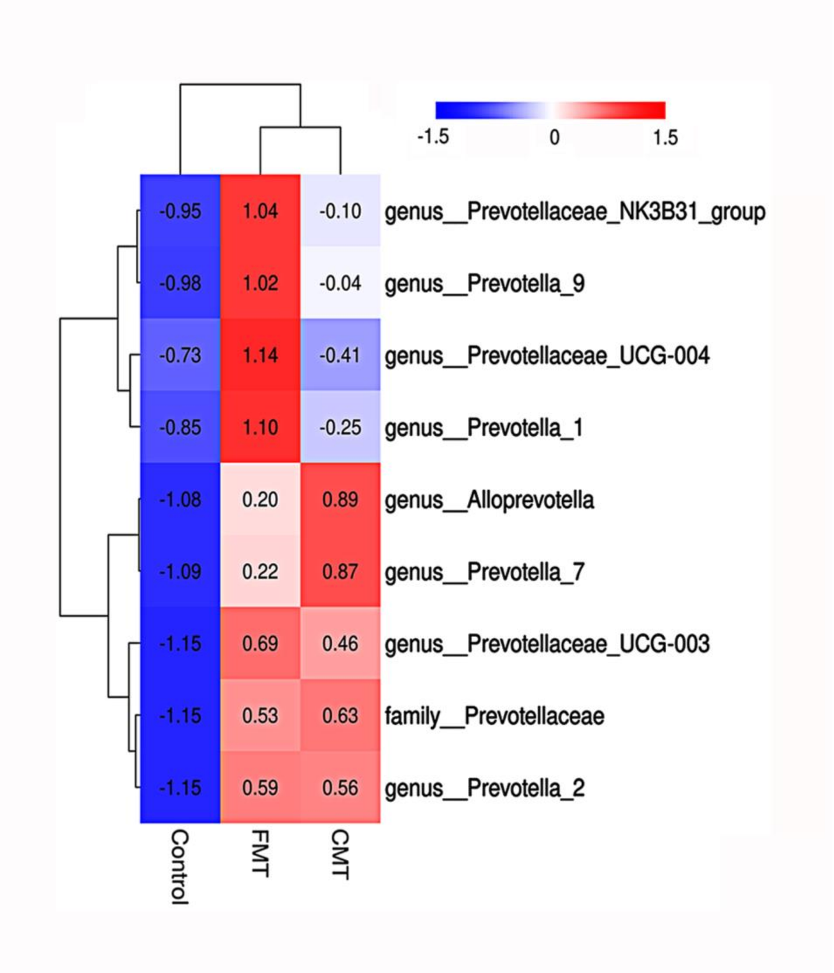

-1.5
0
1.5
-0.95
1.04
-0.10
genus__Prevotellaceae_NK3B31_group
-0.98
1.02
-0.04
genus__Prevotella_9
-0.73
1.14
-0.41
genus__Prevotellaceae_UCG-004
-0.85
1.10
-0.25
genus__Prevotella_1
-1.08
0.20
0.89
genus__Alloprevotella
-1.09
0.22
0.87
genus__Prevotella_7
-1.15
0.69
0.46
genus__Prevotellaceae_UCG-003
-1.15
0.53
0.63
family__Prevotellaceae
-1.15
0.59
0.56
genus__Prevotella_2
Control
FMT
CMT

S Fig.6

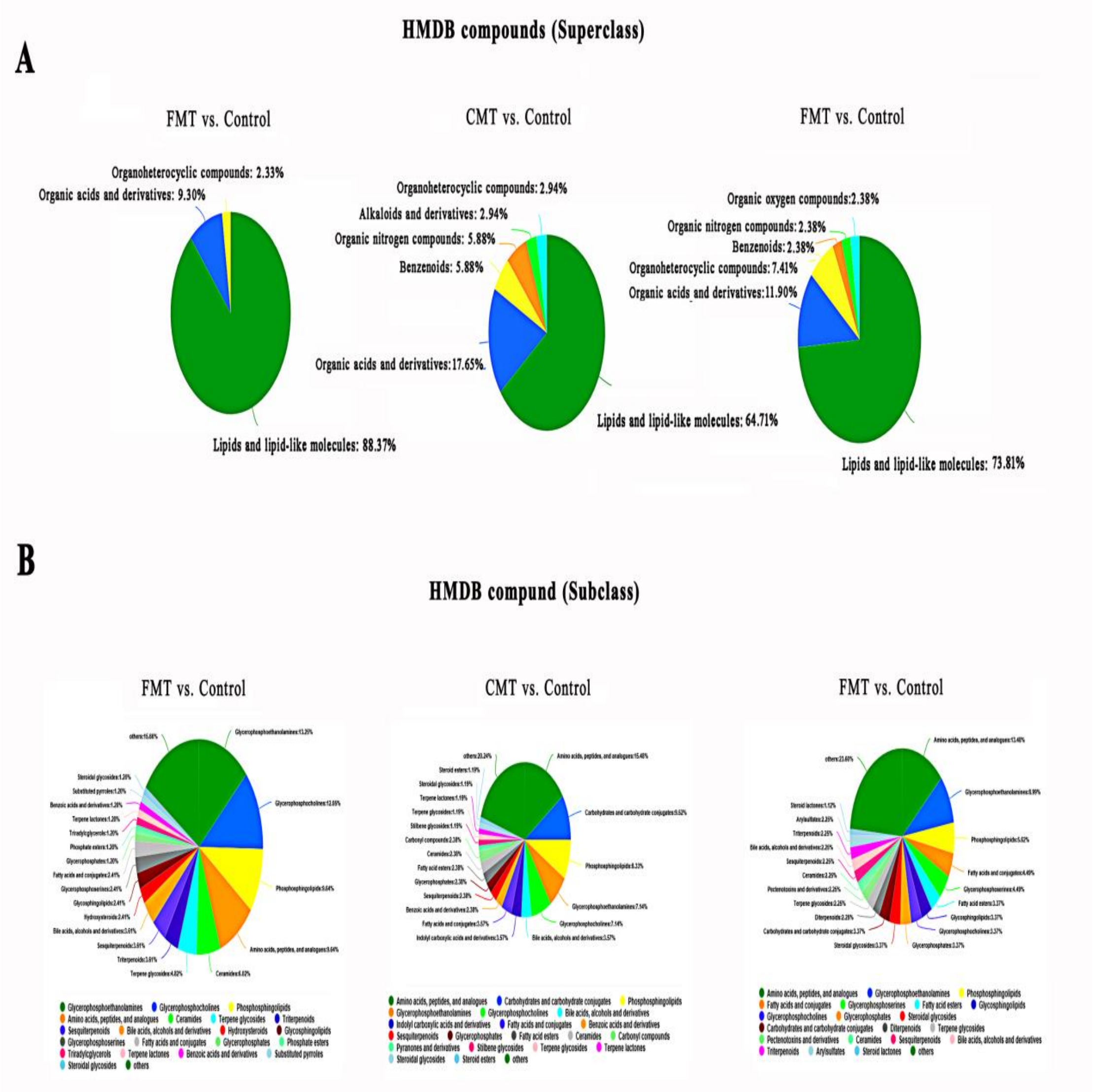
HMDB compounds (Superclass)
A
FMT vs. Control
Organoheterocyclic compounds: 2.33%
Organic acids and derivatives: 9.30%
Lipids and lipid-like molecules: 88.37%
CMT vs. Control
Organoheterocyclic compounds: 2.94%
Alkaloids and derivatives: 2.94%
Organic nitrogen compounds: 5.88%
Benzenoids: 5.88%
Organic acids and derivatives:17.65%
Lipids and lipid-like molecules: 64.71%
FMT vs. Control
Organic oxygen compounds:2.38%
Organic nitrogen compounds: 2.38%
Benzenoids: 2.38%
Organoheterocyclic compounds: 7.41%
Organic acids and derivatives:11.90%
Lipids and lipid-like molecules: 73.81%
B
HMDB compund (Subclass)
FMT vs. Control
CMT vs. Control
FMT vs. Control

S Fig.7

A

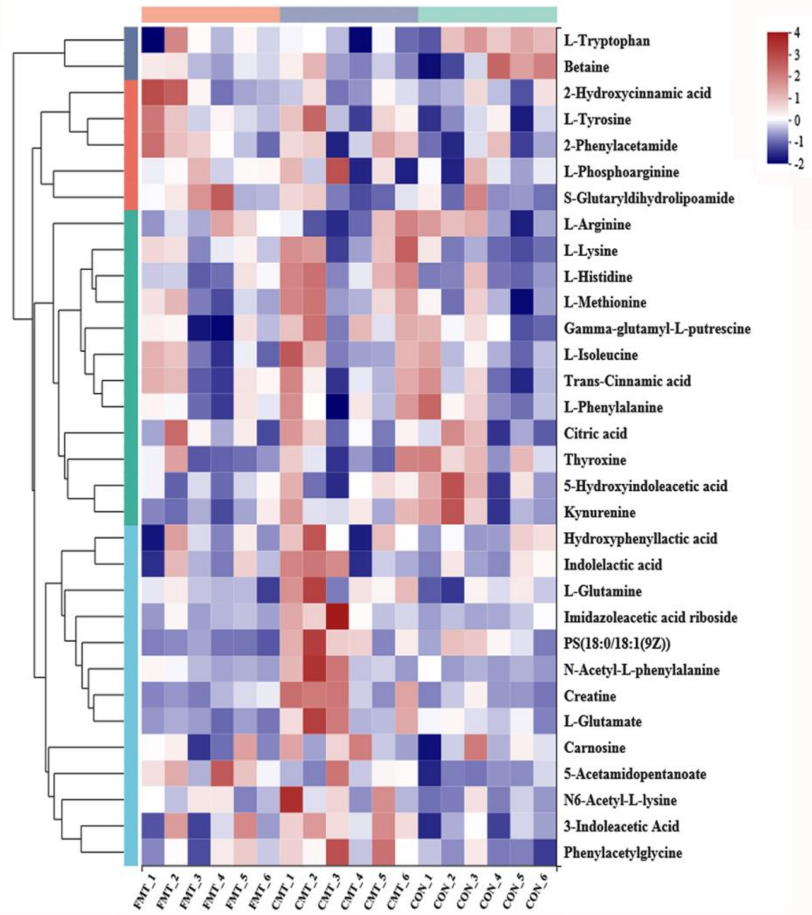

B

Metabolites	Control		FMT		CMT		
	Mean	SEM	Mean	SEM	Mean	SEM	P value
3-Indolepropionic acid	16.81	3.37	20.13	6.31	47.87	14.92	0.0697
3-Indoleacetic Acid	55.70	10.54	84.33	22.41	118.11	11.81	**0.0028**
5-Hydroxyindoleacetic acid	39.83	9.10	21.48	3.90	25.92	7.01	0.2538
Indolelactic acid	172.48	26.11	166.76	44.17	257.75	70.15	0.2812
N-(1-Deoxy-1-fructosyl)tryptophan	9.78	7.18	25.69	23.35	61.61	24.75	0.0720
L-Tryptophan	2763.30	401.77	1912.15	502.94	1442.19	317.80	**0.0275**

S Fig.8
